# *In silico* Analysis of the Binding Affinities of Antigenic Epitopes of Vaccines Administered to Gulf War Veterans to Specific HLA Class II Alleles Protective for Gulf War Illness

**DOI:** 10.29245/2572.942x/2019/5.1254

**Published:** 2019-10-19

**Authors:** Spyros Charonis, Lisa M. James, Apostolos P. Georgopoulos

**Affiliations:** 1 Brain Sciences Center, Department of Veterans Affairs Health Care System, Minneapolis, MN, 5541, USA; 2 Department of Neuroscience, University of Minnesota Medical School, Minneapolis, MN 55455, USA

**Keywords:** Gulf War Illness (GWI), Human Leukocyte Antigen (HLA), vaccines, persistent antigens, immunity, HLA epitope affinity

## Abstract

Gulf War Illness (GWI) is a chronic, multi-symptom disorder of unknown etiology affecting veterans of the 1990-91 Gulf War. We identified previously^1^ a set of 6 Human Leukocyte Antigen (HLA) class II alleles that are protective for GWI, namely DPB1*01:01, DPB1*06:01, DQB1*02:02, DRB1*01:01, DRB1*08:11, and DRB1*13:02. Since the function of HLA class II molecules is to connect with matching extracellular antigens of various pathogens (mostly viruses), as an initial step in the sequence of events leading to the development of antibodies against the matched antigen and its subsequent elimination, we hypothesized that GWI may be due, in part, to the persistence of offending antigens which could not be eliminated^2,3^. We further hypothesized^4,5^ that such antigens were contained in the 16 vaccines administered to GW veterans against adenovirus, anthrax, botulinum, cholera, diphtheria, hepatitis B, influenza A, Japanese encephalitis, measles, meningococcus, poliomyelitis, rabies, smallpox, tetanus, typhoid, yellow fever.^6^ This hypothesis predicts that antigens present in those vaccines should have a high affinity for matching with the 6 HLA class II protective alleles above. Here we tested this prediction by using the Immune Epitope DataBase (IEDB^7^) to determine the ranked affinity of each one of the 6 GWI protective alleles to the 10 most frequently assayed epitopes of each pathogen for which a vaccine was administered. We found that our 6 GWI protective alleles above collectively covered all vaccine antigens except for rubella for which all alleles above showed low binding affinity. Affinity strength varied among antigen-allele pairs, with DRB1*01:01 and DRB1*13:02 showing overall higher affinities. These two alleles also had the highest binding affinities for the anthrax antigen contained in the anthrax vaccine administered to GW veterans. These findings document a good match between the 6 GWI HLA protective alleles above and the antigens contained in the GW vaccines, and support the fundamental assumption that the HLA protection for GWI is mediated through the successful elimination of potentially harmful persistent antigens contained in those vaccines.

## Introduction

Gulf War Illness (GWI) is a disease of unknown etiology that affects several organ systems, including the brain. Patients with GWI show brain atrophy^8^ and functional neural network abnormalities resembling those of immune-related disorders^9^, hence the characterization of GWI as a “neuroimmune” disease^9^. Levels of inflammatory markers, including C-reactive protein (CRP), are increased in GWI^10,11^ and are positively correlated with the severity of GWI symptoms^11^. These findings indicate the presence of inflammation in GWI affecting several organs, since CRP levels were correlated with symptom severity in separate symptom domains^11^. We attributed this chronic inflammation to the presence of harmful, persistent antigens coming from vaccines^12^ or other biological exposures (infectious agents) to which GW veterans were subjected. Given the GWI protection conferred by our 6 HLA class II alleles^1^ (DPB1*01:01, DPB1*06:01, DQB1*02:02, DRB1*01:01, DRB1*08:11, DRB1*13:02), we investigated in this study the degree to which these alleles would be likely to eliminate antigens contained in the vaccines administered to GW veterans, thus conferring protection from GWI.

The function of HLA class II genes is to provide adaptive immunity and immunological memory following an initial exposure to a specific pathogen, enabling the host to mount an enhanced response to subsequent encounters with that pathogen^13^. The mechanism through which adaptive immunity is established involves cell membrane receptors encoded by HLA class II genes using their tertiary structure characteristics (binding groove) to chemically bind and display pathogen-derived peptides that are recognized by CD4^+^ T helper cell receptors. Subsequently, antibodies specific to the pathogen-based peptide (antigen) are produced by specialized B lymphocytes and immediate response to this foreign antigen can be mounted if it is encountered again. The HLA genes are highly polymorphic, with many different alleles carried by different individuals within a population. We have hypothesized that GWI could be the result of an inadequate immune response to toxic, persistent antigens caused by lack of protective HLA class II molecules^2^. The “persistent antigen” hypothesis^3^ stipulates that pathogenic antigens are present in the blood of GWI veterans, while healthy GW veterans possess protective alleles and thus were able to produce specific antibodies to neutralize the antigens that “persist” in non-healthy veterans. This hypothesis has been supported by a recent study comparing the effects of healthy sera and GWI sera on neural cell cultures^4^.

To gain a broader understanding of the protective effects of the six GWI protective alleles, we sought to investigate their recognition and binding capabilities on a wide array of pathogens. As a starting point in this direction, a set of pathogens for which GWI veterans received vaccinations was compiled and each of the six protective alleles was searched for recognition and binding capacity to antigens of all pathogens using a highly specialized epitope database.

## Materials and Methods

### Data Collection.

Vaccinations were administered to military personnel prior to and during Gulf War service for a wide array of pathogens.^6^ A set of 20 pathogens which were prescribed for military personnel^6^ was compiled. Binding data for HLA class II alleles were found for the following 20 pathogens: adenovirus, anthrax, botulinum, cholera, diphtheria, hepatitis B, influenza A, Japanese encephalitis, measles, meningococcus, mumps, poliomyelitis, rabies, rubella, smallpox, tetanus, typhoid, varicella, yellow fever, and yersinia pestis (plague). For each pathogen, the predictive tools of the IEDB website (www.iedb.org) were used to identify epitope sequences that are recognized by and bind to HLA class II proteins. Search parameters were set as follows for antigens of each of the 20 pathogens which were queried: All epitope types (linear, discontinuous and non-peptidic), all binding assay types (positive, T cell, B cell and MHC ligand assays), only MHC class II alleles, only human hosts and any disease type (infection, allergy and autoimmune). Results were filtered so that only data published in journal articles were considered. Each search yielded a list of epitopes representing antigenic peptide sequences that recognize human class II HLA proteins. For each queried pathogen, the retrieved epitope sequences were ranked according to the number of positive assays that were reported and the 10 sequences with the highest number of reported assays were selected; for cholera and mumps only 5 and 1 sequences, respectively, were returned by the search. The IEDB identification number of these sequences are given in Appendix.

For each epitope sequence predicted to be an HLA binder, the predicted IC_50_ value (half maximal inhibitory concentration, measured in units of molar concentration nM) and percentile rank (unitless) for peptide binding to our 6 MHC molecules was computed using the NetMHCIIpan method^14^. For a queried antigenic epitope, NetMHCIIpan predicts the specific subsequence that an HLA molecule recognizes (binding core) as well as its binding affinity using an artificial neural network-based model. The binding affinity between the HLA molecule and antigenic sequence is determined using a predicted IC_50_ value and a percentile rank. NetMHCIIpan^14,15^ is one of several methods for quantitative prediction of peptide binding to human MHC class II molecules of known sequence that is integrated into the IEDB prediction tool. NetMHCIIpan was preferred over other methods, as it was the only method capable of making predictions for all our 6 alleles, including DQB1*02:02, for which other methods did not provide binding affinity estimates. The percentile rank for each predicted HLA-binding sequence is generated automatically in IEDB by comparing the peptide’s score against the scores of five million random 15-mers selected from the SwissProt database^16^. Smaller percentile ranks indicate higher binding affinity. We used the minimum percentile rank (highest binding affinity) for each allele-pathogen combination as an estimate of the strength of association of a given allele to a specific pathogen.

### Data Analysis.

Standard statistical methods were employed to analyze the data using the IBM-SPSS statistical package (version 23). The minimum percentile rank values for each of the six alleles across all extracted epitopes and HLA-binding sequences from the 20 pathogens were computed to estimate allele sensitivities to this set of pathogens. Hierarchical tree clustering was employed to visualize the grouping of alleles based on the proximities of their estimated best affinities to various pathogen antigens; for this purpose, between-groups linkage and squared Euclidean distance were used as the clustering method and interval measure, respectively.

## Results

The key values extracted from this dataset that quantify binding affinity between HLA molecules and antigenic epitopes are the predicted percentile ranks which indicate the strength of association between an HLA receptor molecule and the recognized antigenic epitope. The minimum percentile ranks (estimated best affinity) for each allele-pathogen pair are given in Table 1.

**Figure 1:**
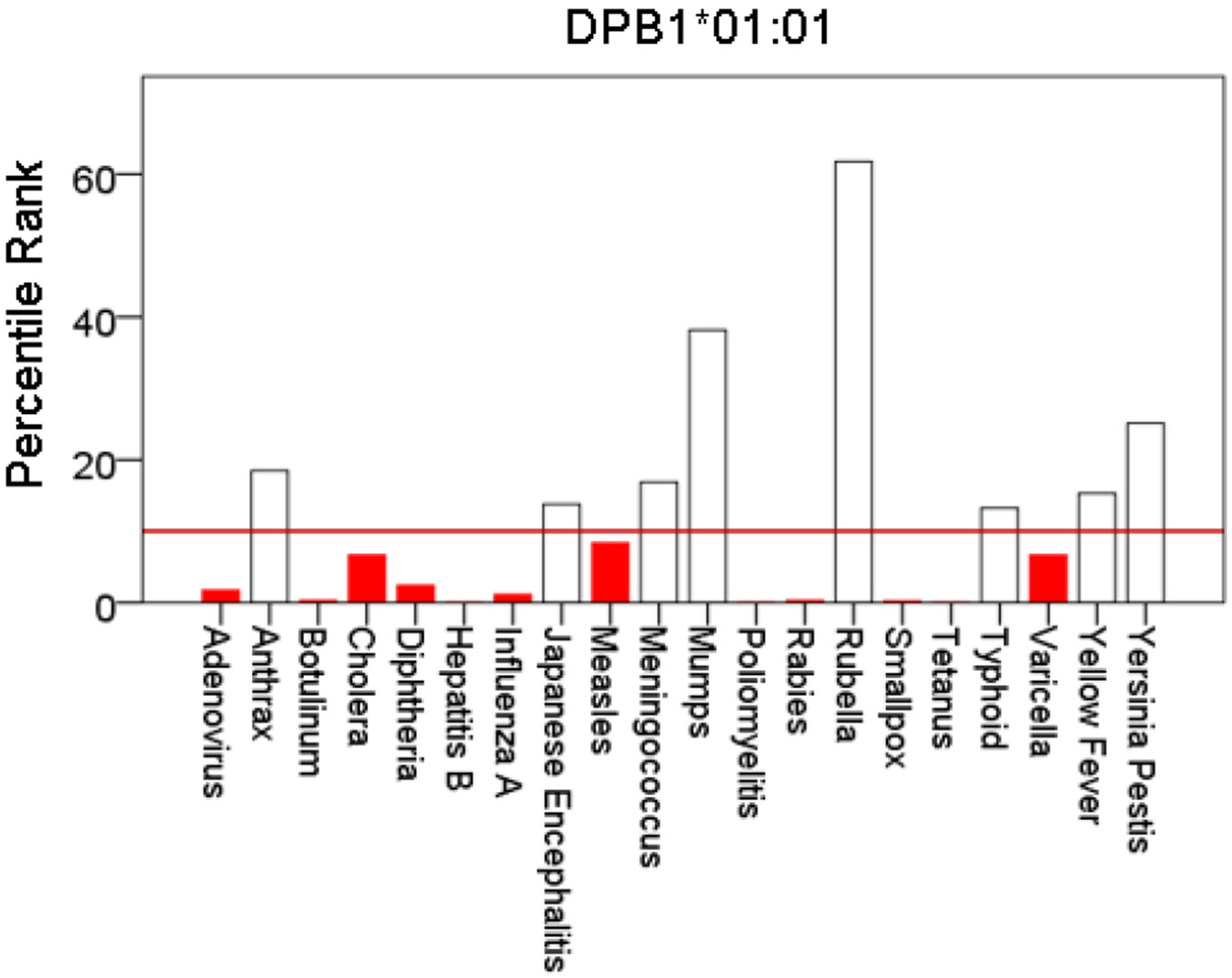
Bars are minimum percentile ranks (best binding affinity) for HLA allele DPB1*01:01 and epitopes of 20 pathogens. Bars in red below the red horizontal line drawn at the 10^th^ percentile indicate good binders. See text for details.

**Figure 2:**
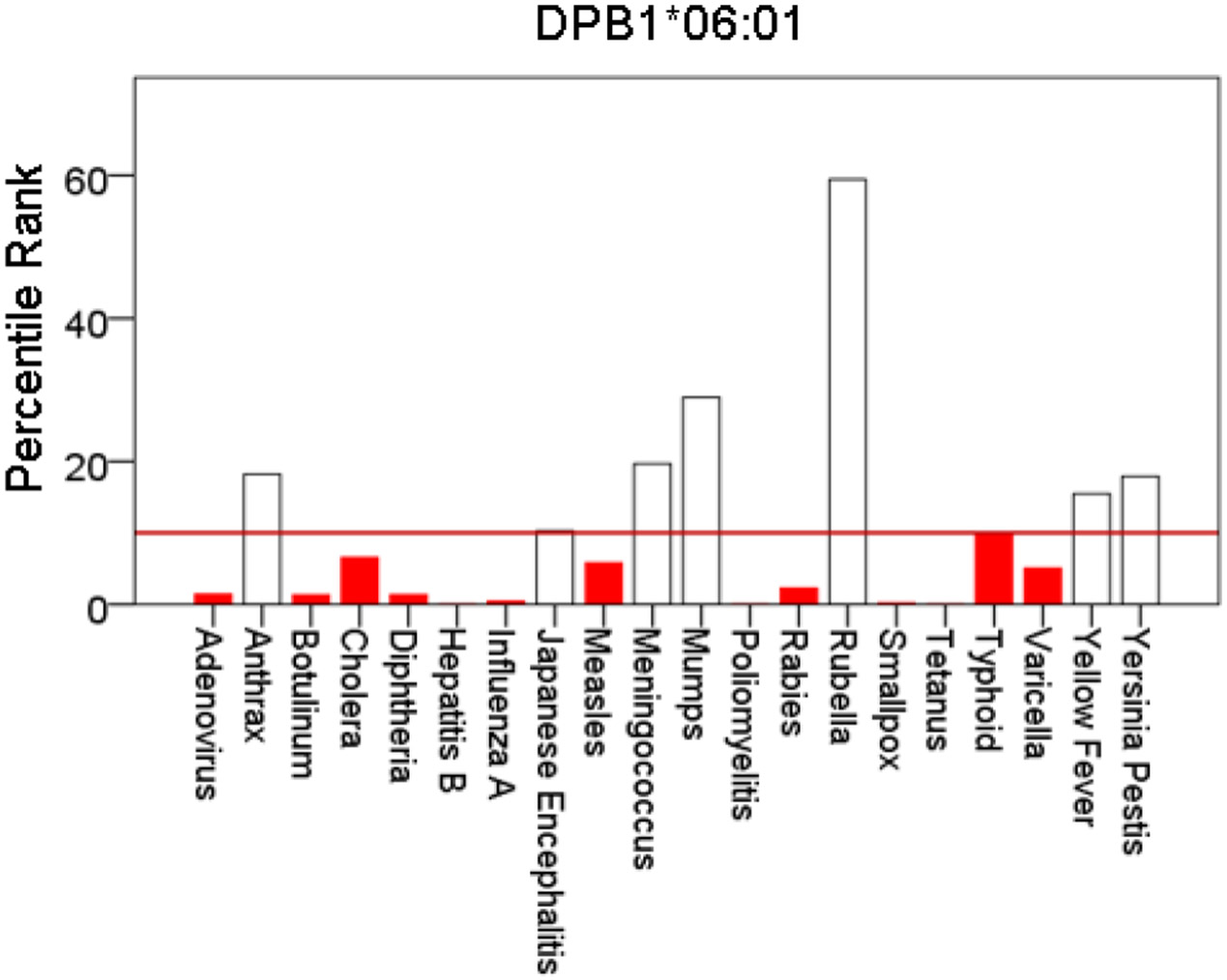
Bars are minimum percentile ranks (best binding affinity) for HLA allele DPB1*06:01 and epitopes of 20 pathogens. Bars in red below the red horizontal line drawn at the 10^th^ percentile indicate good binders. See text for details.

**Figure 3:**
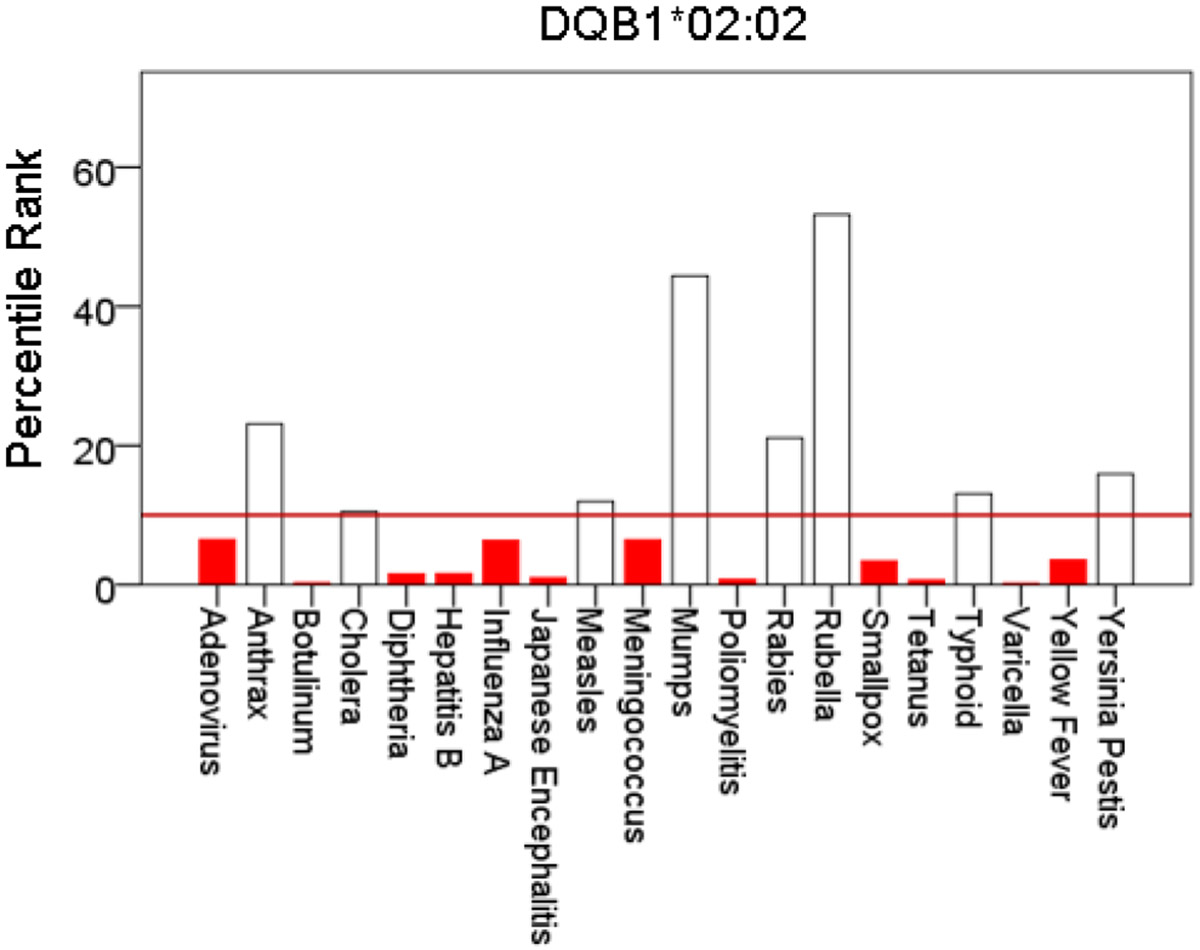
Bars are minimum percentile ranks (best binding affinity) for HLA allele DQB1*02:02 and epitopes of 20 pathogens. Bars in red below the red horizontal line drawn at the 10^th^ percentile indicate good binders. See text for details.

**Figure 4:**
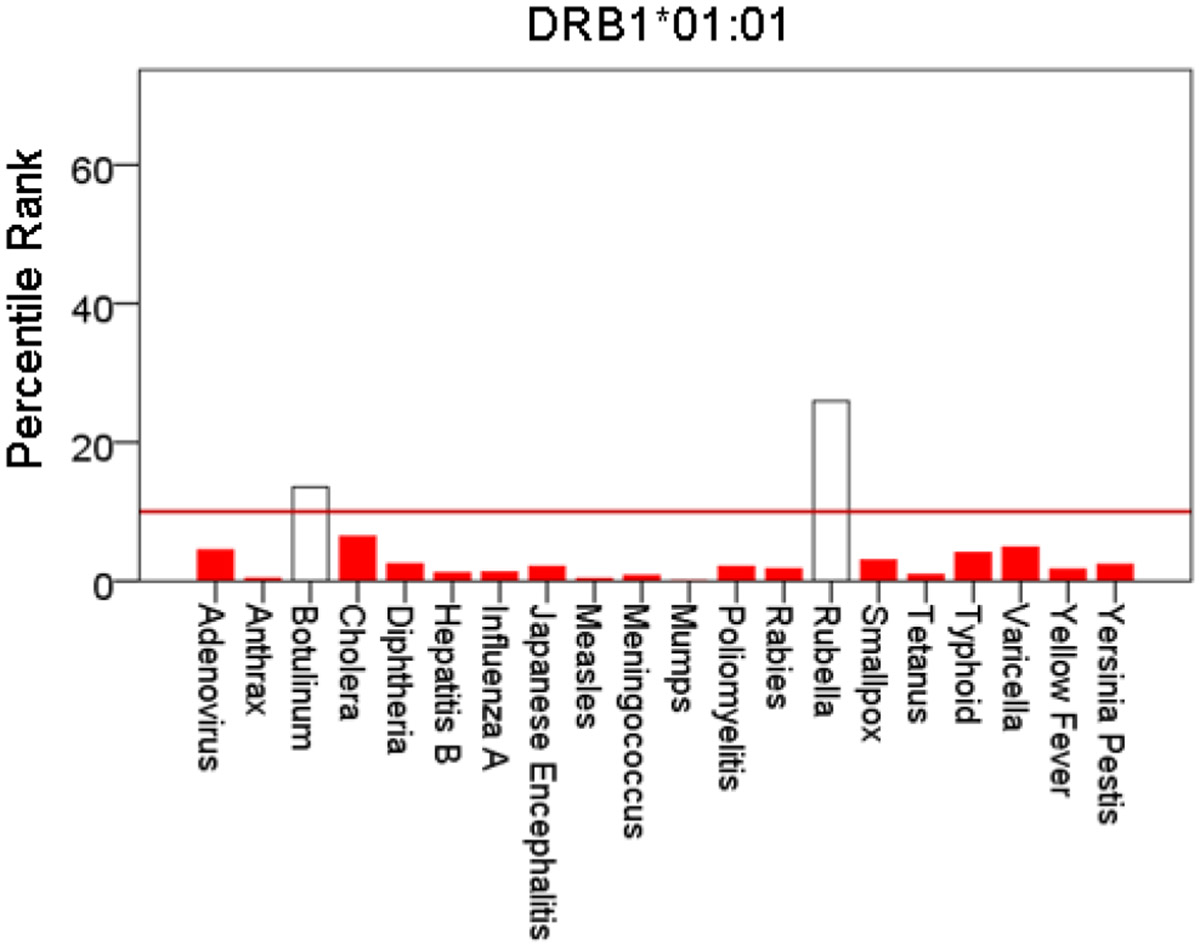
Bars are minimum percentile ranks (best binding affinity) for HLA allele DRB1*01:01 and epitopes of 20 pathogens. Bars in red below the red horizontal line drawn at the 10^th^ percentile indicate good binders. See text for details.

**Figure 5:**
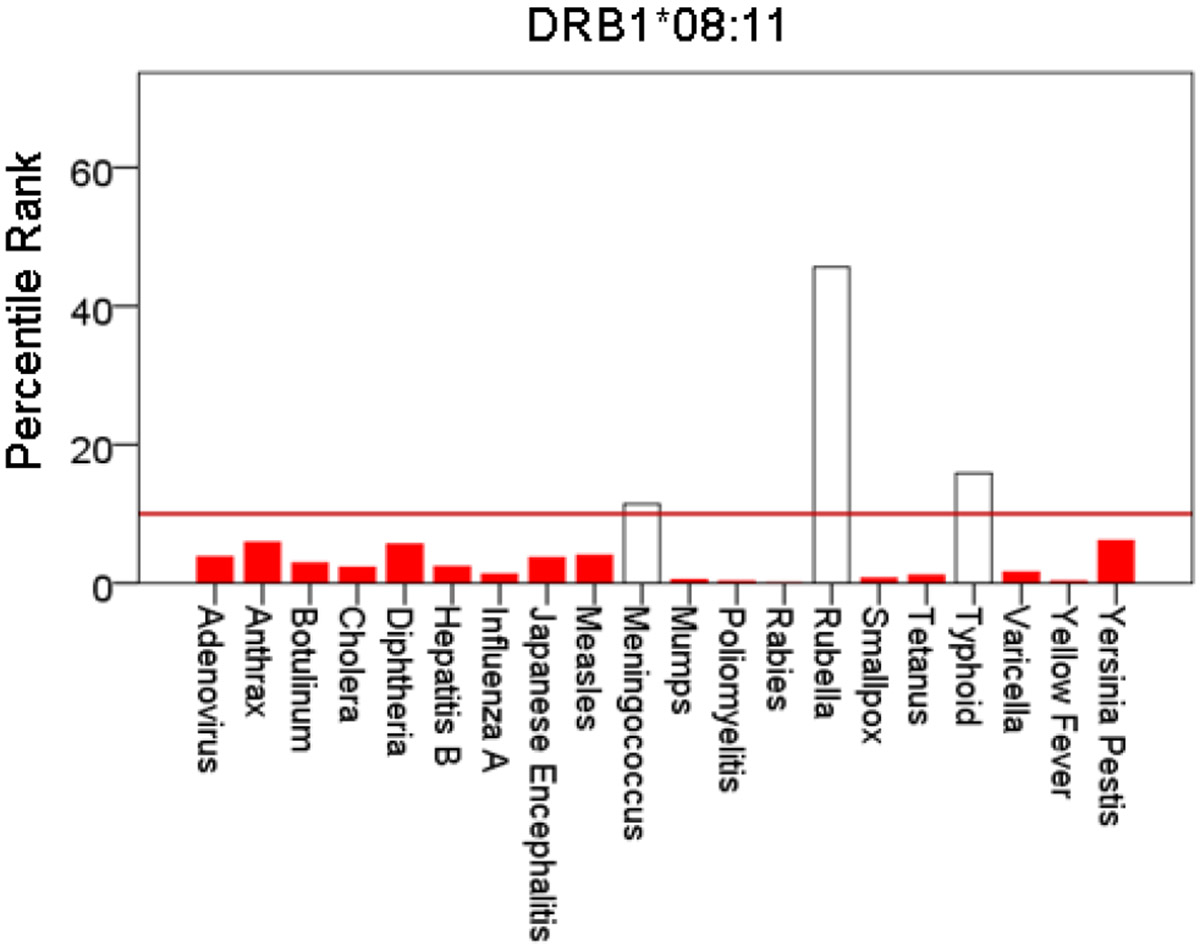
Bars are minimum percentile ranks (best binding affinity) for HLA allele DRB1*08:11 and epitopes of 20 pathogens. Bars in red below the red horizontal line drawn at the 10^th^ percentile indicate good binders. See text for details.

**Figure 6:**
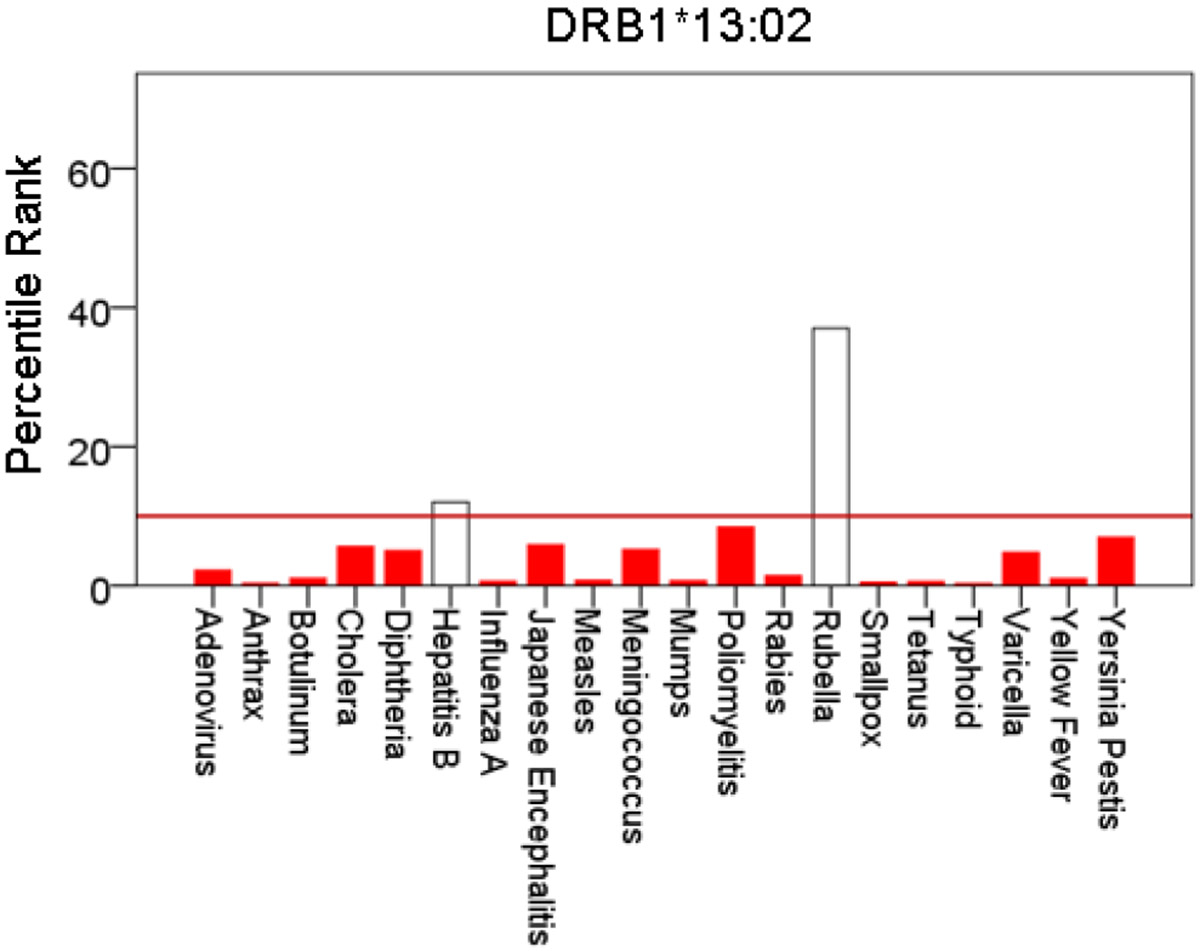
Bars are minimum percentile ranks (best binding affinity) for HLA allele DRB1*13:02 and epitopes of 20 pathogens. Bars in red below the red horizontal line drawn at the 10^th^ percentile indicate good binders. See text for details.

**Figure 7:**
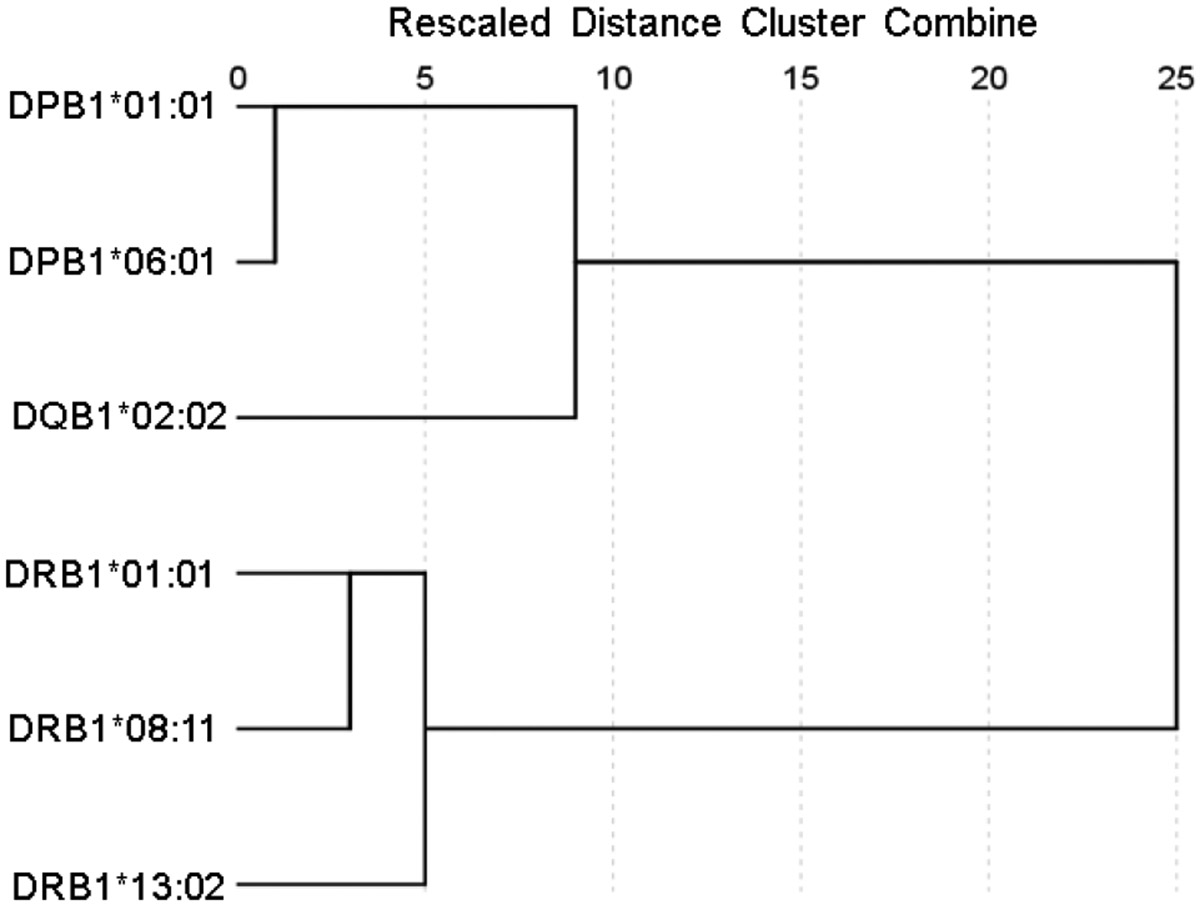
Dendrogram derived from the minimum percentile ranks (Table 1) shows the grouping of the 6 HLA alleles tested to the 3 HLA class II genes to which the 6 alleles belong. See text for details.

Figures 1-6 plot minimum ranks of the 20 pathogens for each allele studied, resulting in 6 “allele-pathogen profiles”. It can be seen that (a) collectively, our 6 GWI-protective alleles possess high affinities for the 20 pathogens, with minimum ranks mostly below the 10^th^ percentile, and (b) more specifically, minimum ranks are, overall, smaller for alleles of the DRB1 gene. In fact, alleles of the different genes were separately associated with respect to their best affinities to the 20 pathogens. The association among different allele-pathogen profiles is illustrated by the dendrogram (tree) of Figure 7 which was derived by applying hierarchical tree clustering on the minimum ranks of Table 1. It can be seen that the tree consists of 2 branches: the upper branch comprises alleles of the DPB1 and DQB1 genes, in separate clusters, whereas the lower branch comprises the alleles of the DRB1 gene, where the DRB1*01:01 and DRB1:13:02 alleles are grouped separately from DRB1*08:11.

**Figure 8:**
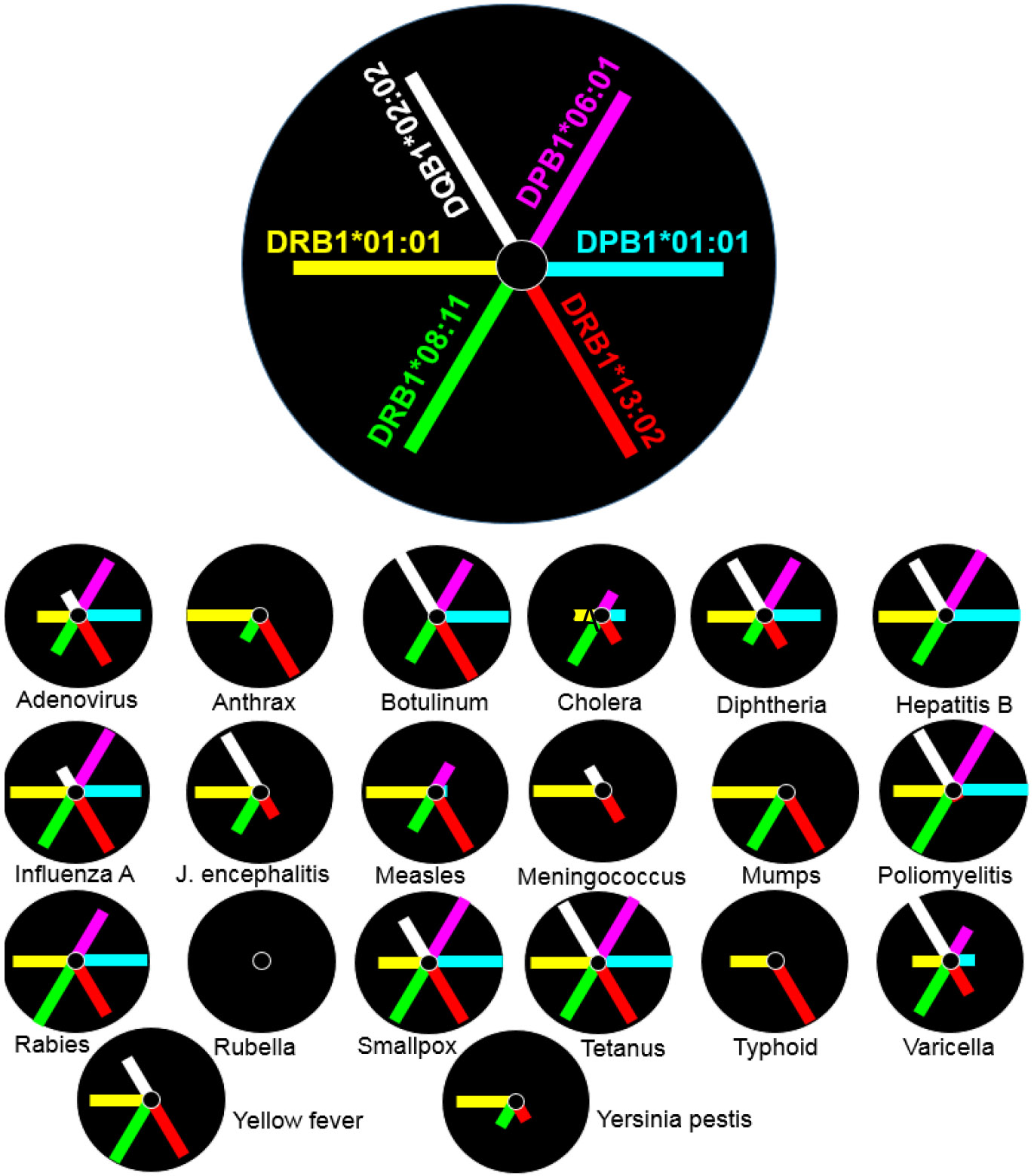
A, position and color coding scheme of the 6 alleles in a radial plot. B, radial plots of allele affinity for each pathogen listed. The radius of the circle is the 10^th^ percentile and the length of the arm is 10 - minmum rank. See text for details.

The distinct allele affinity signatures for each pathogen are illustrated in the radial plots of Figure 8, where the allele is position- and color-coded, as shown in Figure 8A. The radius of the circle in Figure 8B is the 10^th^ percentile minimum rank, (as a reasonable cutoff point for high affinity) and the length of each arm is(10-minimum rank), thus denoting affinity: the longer the arm, the higher the affinity of the allele-pathogen pair. The following can be seen. (a) No allele showed high affinity for rubella; hence the rubella plot is empty; (b) tetanus had a high affinity for all 6 alleles; and (c) all other pathogens had a high affinity for at least 2 alleles.

## Discussion

Here we sought to further understand the protective effects of the six GWI HLA alleles previously identified^1^ by investigating the affinity of those alleles to antigens from vaccines administered to GW veterans. The findings extend our previous research on HLA protection against GWI and suggest that the HLA protection in GWI is conferred through high-affinity binding that promotes antibody production and successful elimination of pathogens. The persistent antigen hypothesis^3^ suggests that, in contrast, veterans lacking the protective alleles are unable to eliminate foreign antigens (including those from vaccines) and that the persistence of those antigens underlies the myriad symptoms associated with GWI due to their pathogenicity. This theory has been supported in recent neural cell culture studies in which GWI sera have been shown to exert deleterious effects that are reduced by the addition of sera from healthy Gulf War veterans^4^ or pooled human immunoglobulin G (IgG)^5^, suggesting that GWI sera are lacking antibodies that are present in healthy GW veterans and IgG. The present findings highlight the influence of HLA makeup in moderating the response to foreign antigens and suggest that exposure to vaccine-derived foreign antigens in veterans lacking HLA protection underlies GWI, providing compelling clues about the nearly 30-year puzzle of GWI.

## HLA Protection

HLA genes, which code for cell-surface glycoproteins, play an essential role in immune protection from foreign antigens by facilitating immune surveillance and initiating an immune response to eliminate foreign antigens. Class I alleles support the elimination of non-specific cytosolic foreign antigens through cell destruction whereas class II alleles facilitate antibody production to specific antigens, promoting adaptive immunity. Antibody production necessitates a match between epitopes derived from foreign antigens and the composition of the HLA receptor binding groove which is determined by HLA genes. Single protein differences alter the binding groove and the landscape of antigens that can bind. That is, each class II allele has a limited repertoire of antigens with which it can sufficiently bind for presentation to CD4^+^ T helper cell receptors to stimulate antibody production. Some class II alleles, however, appear to be more effective than others. Even in the present study, where all 6 of the class II alleles investigated have been shown to protect against GWI, there was considerable variability in binding affinities for the various pathogens. For example, DRB1*01:01 and DRB1*13:02 had a high affinity (≤ 10^th^ percentile) for 18 of the 20 pathogens (90%); DPB1*01:01 and DQB1*02:02 had a high affinity for the fewest number (12 of 20; 60%) of pathogens. Consistent with prior findings that all 6 of these alleles protect against GWI, all were found to bind with high affinity to several pathogens associated with vaccines administered to GW veterans.

The current findings shed light on the mechanism through which the 6 GWI protective HLA alleles exert their effects. In the presence of a match (characterized by high binding affinity) between HLA and a vaccine-derived pathogen, antibodies are created to promote an immune response to subsequent exposure; in the absence of a match, the antigen persists, ultimately leading to inflammation and neurotoxic effects. We have hypothesized that GWI could be the result of an inadequate immune response to toxic, persistent antigens caused by lack of protective HLA class II molecules (i.e., an HLA-antigen mismatch).^2^ Examination of the pathogen-HLA findings here sheds additional light on the types of pathogens that may be associated with GWI.

## Pathogens

Of the 20 pathogens investigated, seven pathogens including adenovirus, diphtheria, influenza A, poliomyelitis, smallpox, tetanus, and varicella matched with high affinity to all 6 protective HLA alleles. Tetanus, in particular, was found to bind with especially high affinity to all of the alleles. An additional five pathogens (botulinum, cholera, hepatitis B, measles, and rabies) matched with high affinity to 5 of the 6 alleles. Taken together, any of the 6 GWI protective HLA alleles facilitate antibody production against the majority of the 20 pathogens investigated. In contrast, Rubella was shown to bind poorly to all 6 of the protective HLA alleles, essentially eliminating Rubella as a causal agent in GWI since these alleles are shown to protect against GWI and none “match” with Rubella. In other cases, pathogens were shown to selectively bind with high affinity to some alleles but not others. Anthrax, for example, binds with very high affinity (< 1^st^ percentile) to DRB1*13:02 and DRB1*01:01, less so with DRB1*08:01, and binds relatively poorly with the other three alleles. Thus, the ability to mount an immune response to these pathogens depends on one’s HLA composition with some alleles exerting broad protective effects against most of the pathogens for which vaccines were administered and other alleles binding with fewer pathogens.

## Summary

Several studies have concluded that vaccinations are associated with GWI^17-19^; however, it has been a challenge to square the fact that only one-third of U.S. Gulf War veterans developed GWI when all were administered routine vaccines in preparation (and during) deployment. HLA composition appears to be a determining factor in the vaccine-GWI association. The present study documents varying affinity between 6 alleles that have been shown to protect against GWI and antigens from vaccines administered to Gulf War veterans. Protective effects of these six alleles appear to be linked to the successful elimination of potentially harmful persistent antigens contained in those vaccines.

## Figures and Tables

**Table 1.** Minimum percentile ranks (best estimated affinity) for all pathogen-allele combinations. The smaller the rank, the higher (better) the binding affinity. A cutoff at the 10^th^ percentile has been taken as a reasonable border to identify good binding (<10) See text for details.

Pathogen	DPB1*01:01	DPB1*06:01	DQB1*02:02	DRB1*01:01	DRB1*08:11	DRB1*13:02	% <10^th^ rank
Adenovirus	1.71	1.43	6.49	4.47	3.75	2.18	100.0
Anthrax	18.49	18.22	23.12	0.36	5.88	0.34	50.0
Botulinum	0.29	1.34	0.26	13.56	2.89	1.07	83.3
Cholera	6.68	6.56	10.52	6.46	2.26	5.61	83.3
Diphtheria	2.42	1.35	1.55	2.44	5.6	5.02	100.0
Hepatitis B	0.01	0.02	1.57	1.16	2.37	12.03	83.3
Influenza A	1.12	0.43	6.37	1.29	1.31	0.63	100.0
J. Encephalitis	13.77	10.31	0.97	2.09	3.67	5.84	66.7
Measles	8.36	5.77	11.98	0.34	3.98	0.76	83.3
Meningococcus	16.87	19.65	6.45	0.8	11.42	5.22	50.0
Mumps	38.18	28.95	44.38	0.03	0.51	0.69	50.0
Poliomyelitis	0.01	0.02	0.77	2.1	0.26	8.41	100.0
Rabies	0.29	2.29	21.11	1.74	0.02	1.42	83.3
Rubella	61.78	59.45	53.21	25.98	45.63	37.01	0.0
Smallpox	0.18	0.14	3.4	3	0.7	0.48	100.0
Tetanus	0.01	0.03	0.67	0.92	1.13	0.58	100.0
Typhoid	13.25	9.99	13.09	4.07	15.87	0.29	33.3
Varicella	6.65	5.03	0.15	4.9	1.6	4.75	100.0
Yellow Fever	15.29	15.45	3.57	1.68	0.26	1.02	66.7
Yersinia Pestis	25.13	17.88	15.93	2.38	6.12	6.96	50.0
% <10^th^ rank	60.0	65.0	60.02	90.0	85.0	90.0	

## Appendix

Identification numbers (ID) of IEDB epitopes retrieved for specific pathogens (see text for details).

Adenovirus: 8127, 17529, 22612, 25708, 26655, 37152, 52862, 54987, 67917, 68067.

Anthrax: 48019, 54845, 79757, 79956, 80549, 81917, 90856, 96266, 96865, 165619.

Botulinum: 2772, 4528, 6650, 9863, 10975, 11729, 11800, 13471, 14730, 15900.

Cholera: 28896, 156243, 156332, 156361, 156457.

Diphtheria: 23040, 50859, 52336, 67981, 68857, 97893, 97898, 97931, 97979, 98054.

Hepatitis B: 15849, 48757, 39347, 70929, 61746, 66038, 66309, 1505, 41238, 58034.

Influenza A: 6795, 6798, 58177, 58375, 65389, 69270, 97597, 178160, 188723, 188765.

Japanese Encephalitis: 116289, 178024, 539068, 539105, 539138, 539162, 539184, 539194, 539197, 539292.

Measles: 4375, 12574, 24640, 37178, 37233, 39958, 40581, 77778, 77804, 87222.

Meningococcus: 1489, 10730, 20282, 23702, 34771, 34772, 66503, 70463, 76160, 127607.

Mumps: 874634.

Poliomyelitis: 31814, 48785, 58511, 59797, 66978, 71769, 79155, 79272, 79436, 79443.

Rabies: 2927, 6545, 7572, 20735, 23111, 23874, 154303, 154920, 155089, 156139.

Rubella: 3704, 21085, 23937, 25195, 36146, 39232, 43373, 45612, 67872, 67896.

Smallpox: 62525, 146164, 146190, 146212, 146262, 146297, 146388, 146391, 146441, 146467.

Tetanus: 5407, 14562, 17207, 30742, 35768, 36647, 39248, 44284, 46701, 52930.

Typhoid: 189947, 189959, 189992, 190012, 232620, 232623, 232630, 232635, 232641, 426692.

Varicella: 1897, 2766, 4252, 18998, 19727, 25923, 40979, 52183, 66363, 70312.

Yellow fever: 152403, 152541, 152596, 152639, 152644, 152679, 152698, 152764, 152942, 190399.

Yersinia pestis (plague): 7887, 8268, 19899, 41851, 43064, 45121, 65115, 70067, 137373, 137376.
